# Advancing code sharing in the computational biology community

**DOI:** 10.1371/journal.pcbi.1010193

**Published:** 2022-06-02

**Authors:** Lauren Cadwallader, Feilim Mac Gabhann, Jason Papin, Virginia E. Pitzer

**Affiliations:** 1 PLOS, San Francisco, California, United States of America; 2 Department of Biomedical Engineering and Institute for Computational Medicine, Johns Hopkins University, Baltimore, Maryland, United States of America; 3 Department of Biomedical Engineering, University of Virginia, Charlottesville, Virginia, United States of America; 4 Department of Epidemiology of Microbial Diseases and Public Health Modeling Unit, Yale School of Public Health, New Haven, Connecticut, United States of America

On March 30, 2021, a new code sharing policy was introduced at *PLOS Computational Biology* [1]. This policy requires any code supporting a publication to be shared unless there are ethical or legal restrictions that prevent sharing. The policy was introduced in response to community desire for a stronger position on code sharing to reflect the fact that the majority of the community already voluntarily share code [2,3]. This community-driven support for open science practices aligns well with the PLOS mission, and, therefore, the implementation of the new policy was a logical progression for the journal. The policy focuses on increasing code sharing as its primary aim, which, in turn, will support reproducibility, and so is not prescriptive to authors about how or where to share their code. The policy (https://journals.plos.org/ploscompbiol/s/code-availability) allows authors to comply in ways which work for them. By the end of the first year of the policy, we expected to see an increase in code sharing rates (the percentage of published research articles that share code) without any negative impact on the publishing demographics or the author, editor, and journal staff experiences. This Editorial reports on the impact of policy over the first 12 months, provides a longitudinal view of code sharing in the journal since 2019, and articulates how this effort can move forward to enhance further sharing, reproducibility, and openness.

## Analysis

PLOS worked with DataSeer (https://dataseer.ai) and Artificial Researcher (https://artificialresearcher.com) to undertake an analysis of research articles published in *PLOS Computational Biology* from the start of January 2019 to the end of March 2022. Code generation and sharing was detected in articles using Natural Language Processing, which examined the text in the Data Availability Statement and the titles and captions found in the “Supplementary Materials” text segment. The code sharing policy was introduced for all research articles submitted after March 30, 2021, so a lag is expected before increased code sharing can be observed in published articles.

According to the DataSeer analysis, more than 99.5% of research articles published between 2019 and 2022 generated code that supported the research presented in the article and thus could in principle be shared. The rate of code sharing for research articles published in *PLOS Computational Biology* before the policy was implemented was 53% in 2019 and 61% in 2020. This rose to 73% for research articles published in 2021, which includes a high proportion of research articles that were submitted before the policy was implemented (83% of articles published in 2021 were submitted before March 30, 2021). Segmenting the published articles to only those that were submitted after the introduction of the policy, the code sharing rate is 87%.

**Fig 1:**
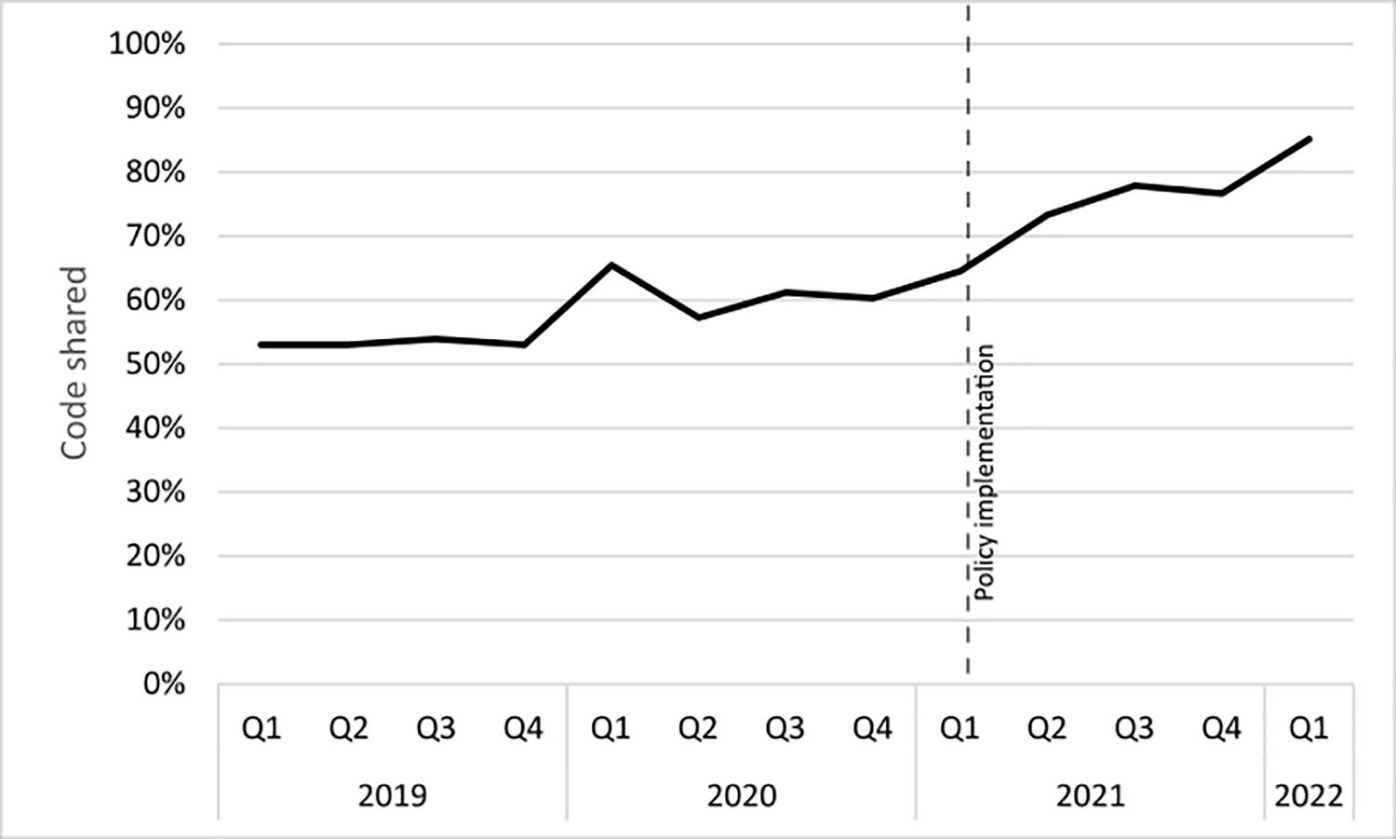
Code sharing rates (% of research articles that share code) of *PLOS Computational Biology* articles, based on the time of publication.

In the years prior to the policy introduction, the data show organic year-on-year increases in code sharing of 8% (2019 to 2020) and 12% (2020 to 2021). This latter increase does include some articles that were submitted after the policy introduction. As the percentage of published articles affected by the policy increases (from 17% of 2021 publications (*n* = 890) to 87% of 2022 publications (*n* = 202)), so does the sharing rate. The increase seen from 2021 to 2022 was 13% (see Fig 1).

## Discussion

The high level of code sharing seen in *PLOS Computational Biology* articles since the policy was introduced is a testament to the community’s openness in research, which is something to be celebrated. Calls for the sharing of code related to research publications are increasing; for example, see current policies from UNESCO [4], the European Commission via Horizon Europe funding scheme [5], and the French National Open Science plan [6]. However, code sharing policies are still not as common as data sharing policies [7,8]. The computational biology community is a leading example of sharing research methods to enable open science.

The organic increase in code sharing seen before the policy was introduced reflects the direction this community was already taking. PLOS’s decision to implement the mandatory code sharing policy was in essence a community-oriented and community-endorsed approach; in fact, the specifics of the policy and its language were reviewed by many of the members of our Editorial Board of 205 computational biologists before implementation. The policy is structured to bring along those community members who may have been slightly skeptical or unsure of code sharing, to demonstrate that barriers to sharing can be overcome and that this is the norm for their community. Informal feedback from our Editors supports these observations. Slightly more than half of Editors responding said that the policy made it easier for them to get authors to share code; the remaining Editors didn’t see a great deal of impact on sharing, presumably due to the willingness of authors to share it voluntarily.

There is still room for improvement—in supporting more authors to share code and in promoting good practices in code sharing. Authors currently not sharing code need to be educated as to what code is expected to be shared and that the default should always be to share the code. Editors and the journal teams also need to ensure that no articles are slipping through the cracks. For those still new to sharing code or unsure what is required at *PLOS Computational Biology*, we want to ensure our support is meeting their needs. The policy is nonprescriptive about how to share code—around two-thirds of authors are sharing via Github, which currently appears to be the community’s preferred sharing method [9]. Authors are expected to share the code that is directly related to findings presented in their manuscript, however trivial or untidy—as Barnes [10] put it: “if your code is good enough to do the job, then it is good enough to release—and releasing it will help your research and your field.”

Of course, code sharing is just one part of open science practices. We encourage all authors to strive toward publishing reproducible research, and we are making headway on how the journal can support more reproducible research in computational biology [11]. Authors who aren’t familiar with this can start by following best practices for code sharing, for example, depositing archived copies in open-access repositories, such as Zenodo; providing clear documentation on how to run the code in the correct environment; and clear licensing of their code (see, for example, [12–15]).

## Next steps

On the back of the results of the first year, the *PLOS Computational Biology* code sharing policy will remain the same, and research has confirmed that a policy-based approach to increase code sharing is most appropriate for the journal currently. [9]. We will continue to monitor the code sharing rates, the submissions we receive to each section, and any issues or barriers that are reported by authors. We will also start looking at issues of code quality and reproducibility and how we might be able to quantify this. The journal, and PLOS more broadly, will continue to provide resources around code sharing for those who need them and encourage best practices to support the community as we all move forward toward fully open and accessible research.

## Data and code availability

The dataset analyzing the code sharing rates at *PLOS Computational Biology* is available from the Figshare repository: http://doi.org/10.6084/m9.figshare.19738846. The code used to create the dataset is also available from the Figshare repository: http://doi.org/10.6084/m9.figshare.19738876.
